# Cardiac Affection in Type 1 Diabetic Patients in Relation to Omentin

**DOI:** 10.3889/oamjms.2015.132

**Published:** 2015-12-13

**Authors:** Soha M. Abd El Dayem, Ahmed A. Battah, Amal El Shehaby

**Affiliations:** 1 *Pediatrics Department, National Research Centre, Cairo, Egypt*; 2 *Critical Care Department, Cairo University, Cairo, Egypt*; 3 *Cairo University, Medical Biochemistry, Cairo, Egypt*

**Keywords:** Cardiovascular, autonomic neuropathy, coronary calcification, type 1 diabetic patient, 24 hr holter

## Abstract

**AIM:**

To evaluate cardiac affection in type 1 diabetes in relation to Omentin.

**PATIENTS AND METHODS:**

Sixty two diabetics and 30 volunteer of the same age and sex were included as a control group. Blood sample was taken for assessment of omentin and oxidized low density lipoprotein (OxLDL), glycosylated hemoglobin (HbA1) and lipid profile. Urine sample was taken for assessment of albumin/creatinine ratio. 24 hour holter was also done. T-test, simple correlation followed by stepwise multiple regression analysis was used for analysis of data.

**RESULTS:**

Parameters of 24 hour holter were significantly lower in diabetics. Omentin was significantly lower, while OxLDL were significantly higher than controls. RMSSD, ST deviation and OxLDL were the parameters related to omentin by stepwise multiple regression analysis in diabetics.

**CONCLUSION:**

Diabetic patients had a cardiac autonomic neuropathy. A significant reduction of omentin and elevation OxLDL imply that they influence glucose metabolism in type 1 diabetes. Omentin had a significant relation to 24 hr holter which may reflect its role in cardiac affection. Omentin and OxLDL had a role in renal affection.

## Introduction

Like an endocrine organ, adipose tissue secretes a variety of adipokines, including leptin, adiponectin, visfatin, TNF-a, and IL-6 [1, 2]. These adipokines have widespread effects on carbohydrate and lipid metabolism and appear to play an important role in the pathogenesis of insulin resistance, diabetes, atherosclerosis, vascular endothelial dysfunction, and inflammation [3–7]. Omentin is a novel fat depot-specific adipokine that was identified from a cDNA library from visceral omental adipose tissue by Yang et al., [8]. The omentin gene is located in the 1q22–q23 chromosomal region, which has been linked to type 2 diabetes in different populations [9–12]. Omentin mRNA is predominantly expressed in the stromal vascular fraction of visceral adipose tissue and is barely detectable in subcutaneous fat depots and mature adipocytes. There are two highly homologous isoforms of omentin, omentin-1 and omentin-2; omentin-1 is the major circulating form in human plasma [13]. The biological activity of omentin is not well understood. Recombinant omentin enhances insulin-stimulated glucose uptake and Akt phosphorylation in human subcutaneous and visceral adipocytes in vitro, but has no effect on basal glucose uptake [14, 15].

We are aiming to evaluate cardiac affection in type 1 diabetic patients in relationship to omentin.

## Patients and Methods

### Patients

The study included 62 patients with type 1 diabetes mellitus (DM) among those attending to the endocrine clinic. The control group consisted of 30 healthy friends and relatives of our patients of age and sex matched. Inclusion criteria: Patients with Duration of disease > 5 years and Patients age > 14 and < 19 yrs old. Exclusion criteria were as follows: Patients during acute diabetic complications e.g. diabetic ketoacidosis (DKA) or hypoglycemia and patients suffering from cardiac diseases, for example, congenital, rheumatic heart, and left ventricular dysfunction and patients receiving drugs for cardiovascular disease.

The study design and protocol was a cross sectional observational study done after obtaining approval from the Ethical Committee of the National Research Centre. The registration number was 11052. Written informed consent was obtained from all patients, their parents and controls after full discussion about the aim of the study. The study conforms to the Declaration of Helsinki. This study is a part of a project undertaken in the National Research Centre for evaluation of cardiac, vascular, and endothelial function in adolescent type 1 diabetic patients. All the studied patients were subjected to history taking including age of patients, sex, age of onset of diabetes, duration of diabetes, type and dose of insulin therapy, and family history of diabetes. We asked about presence of any symptoms of cardiac, renal, neurological affection or presence of any type of autonomic dysfunction. We also asked about history of taking drugs other than insulin. Patients and controls were subjected to general, cardiac, chest, and neurological examination.

Patients and controls were subjected to all methods:

Blood pressure was measured three times after 5-minute rest in the sitting position on both upper limbs with the use of automatic manometer (Omron M4 Plus, Omron Healthcare Europe, Hoofddorp, and Holland). The mean value of the second and the third measurement was calculated. The measurements taken on the dominant limb were analyzed.

Anthropometric measurements in the form of weight, height, waist circumference (WC), and hip circumference (HC) were taken for each participant. The weight and height of the participants were measured up to 0.01 kg and 0.1 cm using a Seca Scale Standing Balance and a Holtain Portable Anthropometer (Holtain, Ltd, Crymmych, Wales, U.K.). Body mass index (BMI) was calculated as weight (in kilograms) divided by height (in meters) squared. Waist circumference was measured at the level of the umbilicus with the participant standing and breathing normally; hip circumference was measured at the level of the iliac crest, using non stretchable plastic tape to the nearest 0.1 cm. The waist / hip ratio and waist/height ratio (cm/cm) were calculated. Each measurement was taken as the mean of three consecutive measurements, using standardized equipment [16, 17]. The landmarks, instruments used, and techniques followed were those recommended by the international biological program [16, 17].

All patients and controls underwent the following tests: For cholesterol measurements, venous blood was sampled after a 12-h fast. Serum total cholesterol was determined by a commercial kit (Boehringer-Mannheim, Germany) [18]. High-density lipoprotein (HDL) cholesterol was separated from the serum by precipitation of the other lipoproteins with a heparin/manganese procedure [19]. Low-density lipoprotein (LDL) cholesterol was calculated using the Friedewald equation. The concentration of triglycerides(Tg) was measured in a TechnoCon AutoAnalyzer II (TechnoCon Instruments, Tarrytown, NY, USA). Glycosylated hemoglobin (HbA1) was done every 3 months and the mean value was calculated per year. It was measured using high pressure liquid chromatography (Nichols Institute, Van Nuys, CA, USA) [20]. Screening for microalbuminuria was assessed in fresh morning urine samples by measuring albumin/creatinine ratio by enzyme linked immunosorbent assay (ELISA) kit provided by Orgentec Diagnostika, Gmbh (Mainz, Germany) [21].

Omentin, protein levels were measured by using commercially available enzyme-linked immunosorbent assays (ELISA) from Adlitteram Diagnostic Laboratories (U.S.A.) according to manufacturer’s protocol.

Serum concentrations of oxidized low-density lipoprotein (OxLDL) were detected by a commercially available solid phase two-site enzyme immunoassay kit (Mercodia AB, Uppsala, Sweden). Measurements of the OxLDL levels in the sera were performed according to the recommendations of the manufacturer. The intra and interassay coefficients of variations were 5.5% – 7.3% and 4.0% – 6.2%, respectively, and the sensitivity was < 1 mU/L [22, 23].

Heart rate variability (HRV) was measured by computerized analysis of long-term heart rate samples (1024 beats) using 24-hour Holter monitoring space lab boarded in which the software automatically calculates all heart rate indices in a comprehensive and accurate form. Spectral analysis was performed on linearly resample (1 Hz) time series using Welch’s method [24]. Time domain analysis was computed on differing computations of the measurement of the standard deviation (SD) of heart period, based on sinus rate and rhythm (R-R) intervals over time [25, 26]. The time domain analysis of heart rate variability can be further divided into two categories. One category is derived from the R-R intervals, using means and standard deviations of the intervals measured in milliseconds. Measures in this category include the SDRR. The SDRR is the standard deviation of all R-R intervals during a 24-h period. Values for SDRR that are < 50 ms have been associated with sudden cardiac death [27]. The second category of time domain variables is derived from differences between adjacent R-R intervals and includes indices that are independent of circadian rhythms. The PNN50 is the proportion of the total R-R intervals that have differences of successive R-R intervals > 50 ms. SDARR is the standard deviation value of all averaged normal sinus R-R intervals for each 5-min segment in the 24-h recordings.

The RMSSD is the square root of the mean squared differences of successive R-R intervals. Reflecting alterations in autonomic function that are primarily vagally mediated, the PNN50, SDRR, and RMSSD correlate highly with high-frequency power, reflecting parasympathetic modulation [25].

Other time domain variables reflect a mixture of parasympathetic, sympathetic, and other physiologic influences [26, 28]. The end of the T wave was defined as the point of maximal change in the slope as the T wave merges with the baseline. QT interval was corrected (QTc) for heart rate by calculating QTc. QTc was calculate using Bazett’ s equation [QTc = QT interval (ms)/√ (60/heart rate)] [29].

### Statistical Analysis

Statistical analysis was conducted using Statistical Package for Social Science (SPSS) program version 15.0 (Chicago, Illinois, USA). t -test for independent variables was done. Mann Whitney U test was done for data not symmetrically distributed. Pearson or Spearman correlation was also done for omentin. Stepwise multiple regression analysis was also performed to find an association of omentin with Variables which had p value <0.05 in simple correlation analysis (Pearson’s or Spearman).

## Results

The study included 62 patients with type 1 diabetes (31 males and 31 females) and 30 healthy volunteer (15 males and 15 females). All diabetic patients were on intensive insulin therapy regimen. Comparison between demographic, anthropometric and laboratory data of patients and controls were shown in Table 1.

**Table 1:** Comparison between demographic data, anthropometric data and laboratory data of patients and controls

**Variables**	**Patients**		**Controls**		**P-value**
	Mean	SD	Mean	SD	
Age (yrs)	16.32	1.52	16.13	2.63	0.70
***Anthropometric data:***					
BMI (kg/m^2^)	24.91	4.20	24.76	5.67	0.8
Waist circumference (cm)	83.60	9.39	84.78	12.25	0.60
HIP circumference (cm)	91.69	8.37	91.20	11.93	0.80
Waist / hip ratio	0.91	0.06	0.93	0.05	0.20
Waist / height ratio	0.51	0.06	0.52	0.08	0.90
***Blood pressure:***					
Systolic blood pressure (mmHg)	119.35	12.53	118.21	14.42	0.70
Diastolic blood pressure (mmHg)	81.94	9.20	78.57	6.51	**0.05**
***Laboratory data:***					
HBA1 (%)	9.55	1.90	5.43	0.65	**0.0001**
Albumin/creatinine ratio (µg/g creatinine)	78.33	100.65	11.28	4.23	**0.0001**
Total cholesterol (mg/dl)	188.81	63.77	100.54	20.41	**0.0001**
Triglyceride (mg/dl)	103.46	78.29	68.89	28.39	**0.03**
HDL-c (mg/dl)	51.77	20.58	52.21	11.12	0.90
LDL-c (mg/dl)	118.66	47.53	62.50	19.88	**0.0001**
Omentin (ng/ml)	18.7	4.3	26.7	4.4	**0.0001**
OxLDL (mg/L)	17.6	6.5	9.1	3.9	**0.0001**

t-test for independent variables, Mann Whitney u test; OxLDL: Oxidized low density lipoprotein, VLDL : very low density lipoprotein, BMI: body mass index

**Figure 1:**
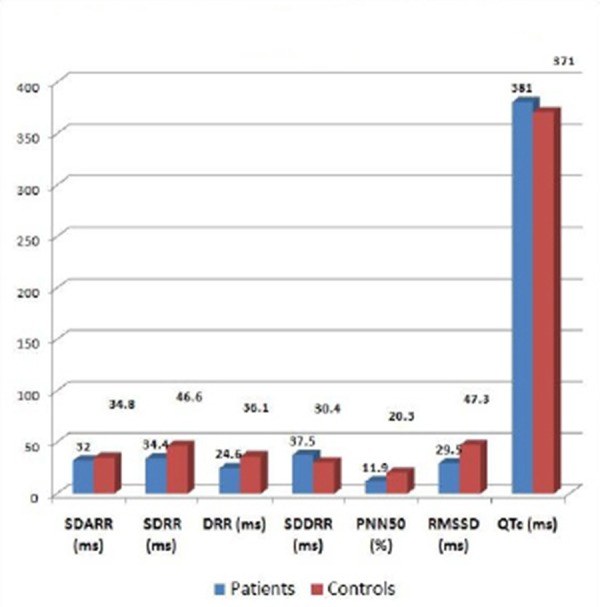
*Comparison between parameters of 24 hr holter of diabetics and controls*.

Omentin was significantly lower, while OxLDL was significantly higher than controls (Table 1). SDRR, DRR, SDDRR, PNN50 and RMSSD were significantly lower in diabetics than controls (Fig. 1). No Significant difference was found between Omentin and sex. Also, no significant correlation was found between omentin and demographic, anthropometric data, HbA1, cholesterol, Tg, HDL and LDL.

Omentin had a significant negative correlation with OxLDL (r = - 0.35, P = 0.01) and VLDL (r = - 0.5, P = 0.02) and positive correlation with RMSSD (r = 0.3, P = 0.04), ST deviation (r = 0.37, P = 0.01).

Omentin had a significant negative correlation (r = - 0.3, P = 0.02), while OxLDL had a significant positive correlation (r = 0.6, P = 0.0001) with albumin/creatinine ratio Table 2).

**Table 2:** Correlation between omentin with demographic, anthropometric, laboratory data and 24 hour holter of diabetic patients

Variables	Omentin	
	r	P-value
***Demographic data:***		
Age of patients (yrs)	0.08	0.56
Duration of disease (yrs)	-0.05	0.71
Insulin dose (U/kg)	0.05	0.69
***Blood pressure:***		
Systolic blood pressure (mmHg)	0.04	0.74
Diastolic blood pressure (mmHg)	0.12	0.36
***Anthropometric data:***		
BMI (SDS)	-0.04	0.75
Waist cirumference (cm)	-0.02	0.86
Hip circumference (cm)	-0.03	0.81
Waist /hip ratio	0.01	0.92
Waist / hieght ratio	-0.04	0.75
***Laboratory data:***		
HBA1 (%)	0.07	0.63
Albumin / creatinine ratio (µg/g creatinine)	- 0.32	**0.02**
Cholesterol (mg/dl)	-0.19	0.16
Triglyceride (mg/dl)	-0.03	0.83
HDL-c (mg/dl)	-0.19	0.14
LDL-c (mg/dl)	-0.12	0.36
VLDL (mg/dl)	-0.54	**0.02**
OXLDL (mg/L)	-0.35	**0.01**
***24 hour Holter:***		
SDARR (ms)	0.20	0.16
SDRR (ms)	0.13	0.35
DRR (ms)	0.05	0.72
SDDRR (ms)	0.01	0.93
PNN50 (%)	0.25	0.06
RMSSD (ms)	0.26	**0.04**
QTC	-0.11	0.44
ST deviation	0.37	**0.01**

Pearson’s or Spearman correlation. OxLDL: Oxidized low density lipoprotein, VLDL: very low density lipoprotein, BMI: body mass index, SDRR is the standard deviation of all R-R intervals during a 24-h period, PNN50 is the proportion of the total R-R intervals that have differences of successive R-R intervals > 50 ms, SDARR is the standard deviation value of all averaged normal sinus R-R intervals for each 5-min segment in the 24-h recordings, The RMSSD is the square root of the mean squared differences of successive R-R intervals. **Bold font indicates significant.**

RMSSD (β = 0.1, 95 % 0.2 -0.04, P = 0.005), ST deviation (β = 3.7, 95 % C.I.: 0.9 – 6.4, P= 0.01) and OxLDL (β = -0.2, 95 % C.I.: -0.4- 0.009, p = 0.04) were the parameters related to omentin by stepwise multiple regression analysis in diabetics (Table 3).

**Table 3:** Stepwise multiple regression analysis of omentin with 24 hr holter, VLDL, albumin / creatinine ratio and oxidized LDL (OxLDl)

Variables	B	95% Confidence interval	P-value
Constant	27.8	21.7 – 33.8	0.0001
RMSSD (ms)	0.1	0.2 – 0.04	0.005
ST deviation	3.7	0.9 – 6.4	0.01
OxLDl (mg/L)	-0.2	-0.4 - -0.009	0.04

R^2^ = 0.5, SEM = 3.43; R^2^: Coefficient of determination, SEM= standard error of mean; Dependent variables: omentin; Independent variables: VLDL, albumin/creatinine ratio, OxLDL, RMSSD and ST deviation; The RMSSD is the square root of the mean squared differences of successive R-R intervals, OxLDL: Oxidized low density lipoprotein

## Discussion

In the current study, HRV in the form of SDRR, DRR, SDDRR, PNN50 and RMSSD were significantly lower in diabetics than controls. Within the pediatric literature, HRV (a measure of cardiovascular autonomic function) was lower in adolescents with type 1 diabetes mellitus (T1DM) compared to healthy controls [26, 30, 31] and lower in youth with T2DM versus T1DM [32]. Chen et al., [33], reported that, time domain is thought to be a marker of parasympathetic function, and these findings suggest that it can be detected early before any manifestation of symptoms and when conventional tests are still normal.

It also suggests that parasympathetic autonomic dysfunction is the first abnormality to arise in the development of cardiac autonomic neuropathy (CAN) [34]. Time domain analysis of HRV, has several advantages over conventional techniques. In addition to being a more sensitive measure of autonomic dysfunction, it is easy to perform with limited specialist training, does not require expensive and cumbersome equipment, is very quick to carry out, and is not affected by subject variability [34].

In the present study, diastolic blood pressure, HbA1, albumin/creatinine ratio, cholesterol, triglyceride, LDL and OxLDL were significantly higher, while omentin was significantly lower in diabetics than controls.

It has been recognized that T1DM is a proinflammatory state and in obese subjects with T1DM, insulin resistance can accelerate progression of T1DM complications [35, 36]. Recently, Tan et al., [37], reported decreased circulating omentin and increased adiponectin levels in subjects with T1DM. Circulating omentin levels and omental adipose tissue (AT) omentin mRNA expression were found to be significantly lower in impaired glucose tolerant (IGT), T2DM subjects and overweight insulin-resistant women with PCOS compared with matched controls [37-40].

Epicardial adipokines, may play an important role in the pathogenesis of coronary vascular disease. In particular, coronary atherosclerosis, given that there is no fibrous fascial layer to hinder diffusion of free fatty acid (FFAs) and adipokines between epicardial AT and the underlying vessel wall as well as the myocardium [37, 41].

In our study, no significant difference was found between Omentin and sex. Also there was no significant correlation between omentin and demographic, anthropometric data, HbA1, cholesterol, triglyceride (Tg), HDL and LDL. In the contrary, Pan et al., [15] and Tan et al., [42], reported that omentin-1 level was lower in female subjects with type 1 diabetes. De Souza Batista et al., [13], reported that, circulating omentin levels were negatively correlated with markers of obesity (body mass index, waist circumference, and circulating leptin).

In the current study, omentin had a significant negative correlation with OxLDL (r = - 0.35, P = 0.01), VLDL (r = - 0.5, P = 0.02), albumin/creatinine ratio (r = - 0.3, P = 0.02) and positive correlation with RMSSD (r = 0.3, P = 0.04) and ST deviation (r = 0.37, P = 0.01). RMSSD (β = 0.1, 95 % C.I.: 0.2 - 0.04, P = 0.005), ST deviation (β = 3.7, 95 % C.I. : 0.9 – 6.4, P 0.01) and OxLDL (β = -0.2, 95 % C.I.: -0.4- 0.009, p = 0.04) were the parameters related to omentin by stepwise multiple regression analysis in diabetics. This means that omentin can be used for early detection of cardiac autonomic dysfunction (microvascular complication) before symptoms of CAN and it is decreased with increase in the oxidative stress. Omentin may play a protective role not only in coronary atherosclerosis but also in other obesity-related cardiovascular disorders, specifically, hypertension, given the vasodilating effect of omentin on blood vessels [35].

Tan et al., [38], found that omentin decreased human sera–induced Akt activation in human endothelial cells. Thus, omentin may play an important role in the relationship between inflammation, angiogenesis, and the pathogenesis of atherosclerosis [43]. Omentin is lower in patients with coronary artery disease [44].

*Limitation of the study:* Number of patients is small, 24 hour Holter takes a long time and needs to convince the patients to use it, Omentin is a new adipocytokines and very minimal lectures are present.

In conclusion, diabetic patients have reduced HRV. Omentin is lower in diabetic patients and this could help for the utilization as a prospective bio marker for cardiac pathology. This novel adipocytokine may be important future targets for the development of drugs and therapy for treating metabolic vascular disorders. Genetic study of omentin polymorphism is essential.
